# Supplementary material for: Magnetic bioassembly platforms for establishing craniofacial exocrine gland organoids as aging in vitro models
Source: PLoS One. 2022 Aug 5;17(8):e0272644. doi: 10.1371/journal.pone.0272644 (PMC9355193; doi:10.1371/journal.pone.0272644)
Supplement: S1 File — This protocol was developed at protocols.io, which can be assessed via this DOI: [https://dx.doi.org/10.17504/protocols.io.b5ttq6nn]. (PDF) [file pone.0272644.s001.pdf]

Establishment of craniofacial exocrine gland organoid magnetic bioassembly platforms as aging multi-omic signatures

Teerapat Rodboon[1], Glauco Souza[2,3,4], Apiwat Mutirangura[5], Joao N. Ferreira[1]

[1]Avatar Biotechnologies for Oral Health and Healthy Longevity Research Unit, Faculty of Dentistry, Chulalongkorn University, Bangkok, Thailand;

[2]University of Texas Health Sciences Center at Houston, Houston, TX, USA; [3]Nano3D Biosciences Inc., Houston, TX, USA;

[4]Greiner Bio-One North America Inc, Monroe, NC, USA;

[5]Department of Anatomy, Faculty of Medicine, Chulalongkorn University, Bangkok, Thailand

1

Teerapat Rodboon
1Avatar Biotechnologies for Oral Health and Healthy Longevit...

In the last decade, relevant biotechnology advances took place in the biofabrication of craniofacial exocrine gland organoids mimicking lacrimal and salivary glands. Though certain challenges still remain not only due to the lack of protocols for organoid reproducibility but also towards the scarcity of methodologies for creating preclinical disease models with aging multi-omic signatures. Previously, our research group successful developed three-dimensional (3D) bioassembly technologies towards the generation of functional epithelial gland-like organoids via magnetic 3D bioprinting platforms (M3DB). To meet the needs of our aging Asian societies, a next step was taken to design consistent M3DB protocols for bioengineering organoid models with aging molecular and pathological features for lacrimal glands (LG) and salivary glands (SG). Herein, we established a feasible step-by-step protocol for producing both LG and SG organoids using M3DB platforms. Such a protocol provided reproducible preliminary outcomes resembling LG/SG organoids. Both acinar and ductal epithelial compartments were prominent (21 4.32% versus 42 6.72% of total cells, respectively), and could be clearly identified in these organoids. Meanwhile, these can be further developed into aging signature models by inducing cellular senescence via chemical mutagenesis. The generation of senescence-like organoids is our ultimate milestone aiming towards high throughput applications for drug screening and discovery and gene therapy investigations to reverse aging.

Teerapat Rodboon, Glauco Souza, Apiwat Mutirangura, Joao N. Ferreira . Establishment of craniofacial exocrine gland organoid magnetic bioassembly platforms as aging multi-omic signatures. **protocols.io**
https://protocols.io/view/establishment-of-craniofacial-exocrine-gland-organ-b5ttq6nn

Thailand Science Research and Innovation Fund Chulalongkorn University
Grant ID: CU_FRB65_hea (7)_013_32_08

Ratchadaphiseksomphot Endowment Fund, Chulalongkorn University
Grant ID: 33/2565 : RU

Postdoctoral Fellowship, Ratchadapisek Somphot Fund, Chulalongkorn University
Grant ID: -

Lacrimal gland, Salivary gland, Xerophthalmia, Xerostomia, Aging, Cellular senescence, Organoids, Bioprinting

Mar 02, 2022

Mar 08, 2022

1 Mechanical and enzymatic primary cell dissociations from craniofacial exocrine glands

Gland dissection and mechanical tissue dissociation

1.1 Lacrimal gland (LG) and salivary submandibular gland (SG) are dissociated from a 3 to 5 month-old porcine head. The head is carried inside an ice-containing box and delivered to the laboratory within 08:00:00 after animal sacrifice. 8h

1.2 Clean the porcine head with sterilized water to remove any debris from the skin before disinfecting it with 0.5 % (v/v) peracetic acid solution for 00:15:00 . 15m

1.3 Gently wash with 1 L of sterilized reverse osmosis (RO) water for 5 times and dry the skin by applying tissue paper.

1.4 Disinfect the skin around the eyes (for the LG) and at the angle of the mandible (for the SG) with 70 % (v/v) ethanol followed by 1 % (w/v) Betadine solution **Contributed by users** and dissect the glands (Figure 1A).

1.5 Collect the glands in a 50 cm Petri dish (Figure 1B) and disinfect with 70 % (v/v) ethanol. To protect the dry out of tissue, covering the gland with 2 mL of collection media.

1.6 Remove peripheral fat and connective tissue by using precision forceps and scissors (Figure 1C) before sectioning the tissue with a scalpel into 0.5 cm3 to 1 cm3 pieces (Figure 1D).

1.7 Transfer tissue into a 50 mL collection tube and wash with 20 mL collection media for 3-5 times or until the solution is clear.

1.8 Keep the tissue in 20 mL collection media at 4 °C to 7 °C for 06:00:00 to 08:00:00 . 14h

> Make sure glands are fully submerged in collection media inside 50 mL tube. Flipping the capped tube upside down is recommended for wetting the gland tissue and minimizing the adherence of the tissue to the lateral walls of the tube.

1.9 Transfer **200 mg** of gland tissue into a glass spot plate and add **500 µL** of collection buffer to prevent tissue to dry out.

1.10 Mince the tissue using a surgical curved scissor into an apple sauce-like slurry paste (Figure 1D) and transfer the slurry into a 15 mL conical tube with a pre-wet 2.5 mL disposable Pasteur pipette.

1.11 Add **10 mL** of washing buffer and pipette up and down by using a pre-wet 10 mL serological pipette.

> Supernatant can become highly viscous in this step due to mucins produced by the glands.

1.12 Let the tissue settle at the bottom of the tube by gravity for **00:03:00** and remove the supernatant using a pre-wet 10 mL serological pipette. 3m

> Small pieces of adipose tissues are float and can be removed at this step.

1.13 Repeat steps **go to step #1.11** to **go to step #1.12** for 3-5 times until the supernatant is clear.

1.14 5m

Centrifuge the tissue slurry at **1000 x g, 00:05:00** and carefully discard the supernatant.

1.15 Add **2 mL** (for 200 mg of tissue) of digestion buffer into a tissue fragment and gently mix well by vortexing. Make sure that no tissue fragment sticks on the lateral walls of the tube after vortexing.

1.16 Wrap the cap and neck of the tube with parafilm to minimize contamination.

1.17 45m

Place the tube into a **37 °C** water beaker with magnetic stirring at 500 rpm and incubate for **00:30:00** (vortex the tube every **00:15:00**).

1.18 Refresh the enzymatic activity by repeating step go to step #1.14 to go to step #1.17 one more time.

> The success of the enzymatic single cell dissociation can be monitored by taking the cell suspension solution and observing the cells under a bright-field microscope. The enzymatic dissociation is completed when clusters of 3-5 cells are observed. The duration of enzymatic digestion varies according to the freshness and fibrous nature of the gland. In case of the digestion is not fully completed, a longer incubation time can be done but avoid going over 2 hours. Longer enzymatic incubation time leads to higher cell yield, but at the expenses of lower cell viability.

1.19 The enzymatic dissociation activity is stopped by a dilution technique.

1.20

Add 8 mL mL of washing buffer into the mixture and gently mix by pipetting for 3-5 times using a pre-wet serological pipette.

1.21 5m

Centrifuge the mixture at 2000 x g, 00:05:00 and discard the supernatant.

1.22 1.2.1Repeat step 1.2.12-1.2.13 for two more times.

1.23 Resuspend the cell pellet by adding 2 mL of washing buffer and mix vigorously with a pre-wet 10 mL serological pipet.

1.24 Transfer the mixture by using the same pipette to the top of a mesh filter (100 µm pore size).

1.25 Wash the serological pipette and mesh filter with an additional 3 mL washing buffer.

1.26 Collect the flow-through cell suspension.

1.27

To make a single cell suspension solution, gently aspirate the flow-through cell suspension by using

a 29 G syringe. Then, gently pass the suspension through a **40 µm** mesh strainer by pressing against the mesh on a circular motion.

1.28

Wash the syringe and mesh strainer with an additional **5 mL** washing buffer.

1.29

5m

Centrifuge the cell suspension at **1000 x g, 00:05:00** and discard the supernatant.

1.30 Carefully aspirate the supernatant by a pre-wet P1000 pipette tip (~300 µL of buffer can be left on the pellet).

1.31 Add **2 mL** of culture media into the cell suspension and gently pipetting with a pre-wet P1000 pipette tip.

1.32

Assess the quality of the primary cells by determining the cell numbers and viability with the Trypan blue exclusion method and can confirm such counts with a

Countess 3 FL Automated Cell Counter
Automated Cell Counter
Thermofisher scientific AMQAF2000

After isolation, the average number of primary cells isolated from 200 mg porcine LG/SG is approximately $0.8x10^6$cells to $1.0x10^6$ cells. The percentage of cell viability should be higher than 80%.

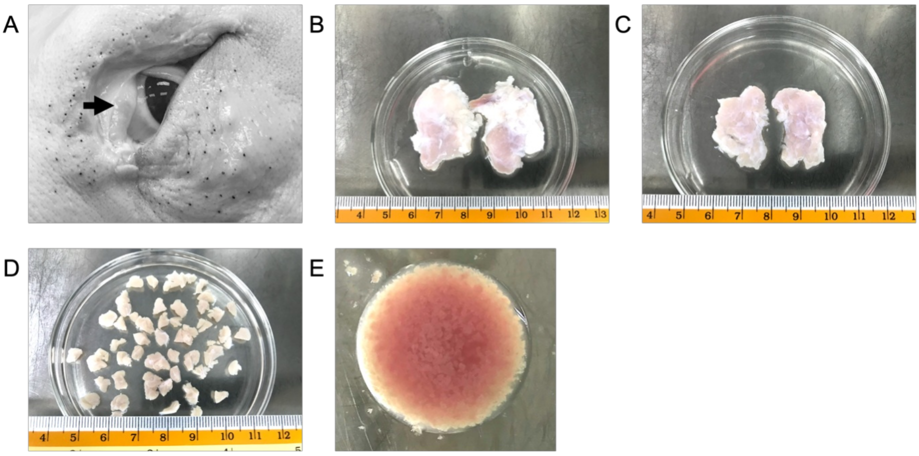

Figure1. Gland specimen preparation. Lacrimal glands (arrow) underneath the superior-lateral portion of the eyelid in each of the porcine orbits (A) were dissected and placed into a Petri dish (B). Encapsulated connective tissue surrounding each gland was removed (C) before cutting the gland into 0.5-1 cm tissue pieces (D). Tissue pieces were then minced into a slurry apple sauce-like appearance before isolating cells using an enzymatic dissociation technique.

Cell plating and culture

1.33 ⚠

One day before cell plating, thaw a vial of Cultrex Reduced Growth Factor Basement Membrane Extract **R&D Systems Catalog #343301001** (BME) overnight in 4°C and use it for coating the growth surface area of a tissue culture flask T75 prior to cell culture.

1.34 For T75 flask coating, pipette **10 µL** of the BME into a 15 mL tube containing **5 mL** of cold serum-free DMEM/F12 **Gibco, ThermoFisher Catalog #21331020** media to make a 1:50 dilution.

1.35 Place a vial of BME on ice during work to prevent untimely gelling.

1.36 Pipetting solution up and down with a 5mL serological pipette by being careful not to create air bubbles and then transfer the mixture into a T75 tissue culture flask.

1.37 Gently swirl the mixture to cover the entire growth surface area and incubate the flask at **Room temperature** for **01:00:00** . 1h

1.38 Remove the mixture after incubation. The flask is ready for cell plating or can be kept in **4 °C** **Overnight** for plating on the following day.

1.39 For cell plating, pipette 1.0x10^5 cells in **10 mL** of expansion media into a BME-coated T75 culture flask.

1.40

Incubate cells at 37 °C 5 % CO_2.

1.41

Observe the morphological heterogeneity (Figure 2) under a light microscope and replace the media every 2 days.

Cells can be passaged at confluency of 70%-80%, which usually occurs 5 to 6 days after plating.

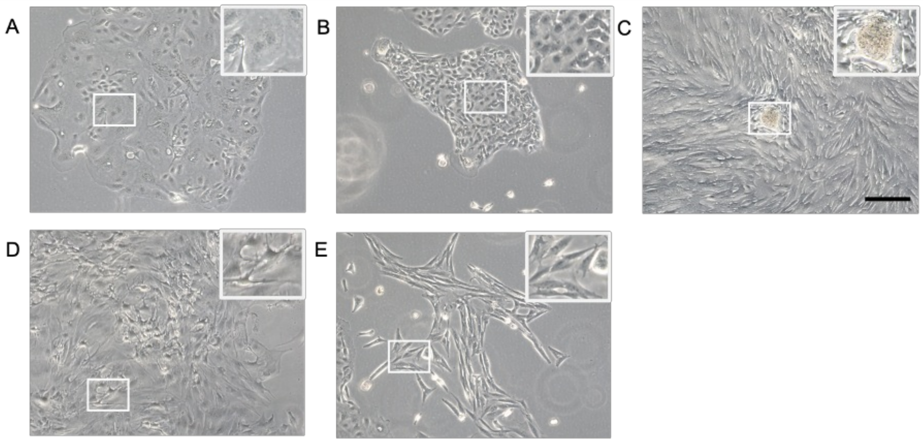

Figure 2. Morphological heterogeneity of primary LG cells. Primary cells isolated from porcine LG are cultured in expansion media for 7 days. Populations of large polygonal-like epithelial cells (A), small polygonal-like epithelial cells (B), epithelial spherule (C), dendritic cells (D), and spindle cells (E) are observed under phase-contrast light microscopy at 20X magnification. Scale bar: 200 µm.

Cell passaging and epithelial enrichment and sorting

1.42 After the confluency of the monolayer cells reached 70%-80%, remove the old expansion media from a culture flask with a 10 mL serological pipette.

1m

1.43 Transfer 10 mL of 1XPBS into the flask and incubate for 00:01:00 .

1.44 Discard the solution from the cells before pipetting 1 mL of TrypLE™ Select Enzyme (1X), no phenol red **Thermo Fisher Catalog #12563011** to cover the monolayer cells.

20m

1.45

Incubate with the enzyme solution at 37 °C for 00:15:00 . To enhance the cell dissociation process, remove the flask from the incubator to swirl or shake every 00:05:00 .

1.46

Observe the single cell dissociation phenomena under a light microscope.

1.47 Stop the enzymatic reaction using a dilution technique: use a 10 mL serological pipette to transfer **9 mL** of basal medium into a flask and resuspend the suspension by pipetting up and down for 3-5 times.

1.48 5m

Transfer the suspension into a 15 mL conical tube and pellet the cells by centrifugation at **1000 x g, 00:05:00** .

1.49 Discard the supernatant and resuspend the pellet by using **5 mL** of expansion media.

1.50

Assess the cell number and viability by using Trypan blue exclusion method and confirm the cell count with

> Countess 3 FL Automated Cell Counter
> Automated Cell Counter
> **Thermofisher scientific AMQAF2000**

1.51

Pipette 1.0x10^5 cells in **10 mL** of expansion medium into a new BME-coated T75 tissue culture flask and incubate the cells at **37 °C** **5 %** CO_2.

1.52 To passage the cells or perform epithelial cell enrichment and/or sorting. Replace the old expansion medium with **10 mL** of fresh EM or EEM at day 2 and replace the medium every **48:00:00** until cells reach the desired confluency. 2d

> To obtain a large cellular heterogeneity, cells should be passage and use for sorting epithelial cells until passage 3 (Figure 3). The population of acinar epithelial cells and ductal epithelial cells can be investigated by determining the expression of AQP5, K14 and K19 protein markers by immunocytochemistry and perform the cell counting with a Countess 3.

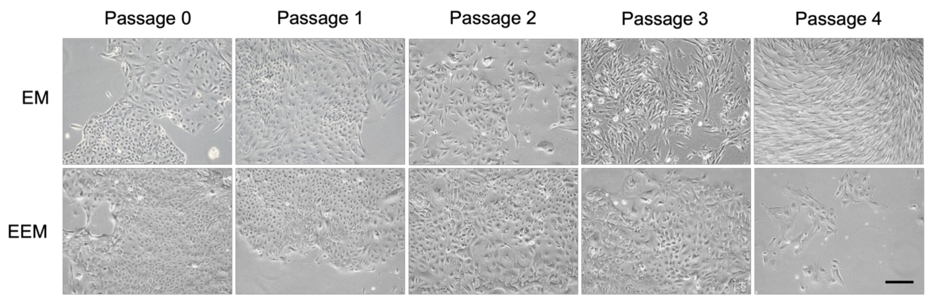

Figure 3. Morphology of primary LG cells in micrographs taken with phase-contrast light microscopy. Primary LG cells culture in expansion medium (EM) and epithelial enrichment medium (EEM) at 7 days for 4 passages. Scale bar: 200 µm.

2 Aging Organoid Establishment with Magnetic Bioassembly

Cell magnetic bioassembly and senescence induction

2.1 2.1.1Before magnetization, dissociate the cells at the monolayer stage at the confluency of 70-80% by following the previous steps in section 1 go to step #1.42 .

2.2 Then, resuspend the cell pellet with 1 mL of epithelial enrichment media (EEM).

2.3

Determine cell numbers and viability by Trypan blue exclusion method and confirm the count with a

Countess 3 FL Automated Cell Counter
Automated Cell Counter
Thermofisher scientific AMQAF2000

.

Cell viability must be greater than 80%

2.4 To fabricate 20 organoids, prepare 420 µL µL of the cells at a density 1.0x10^6 cells/mL in a 50 mm dish by adjusting with EEM.

2.5 Add 42 µL of the NanoShuttle **greiner bio-one Catalog #657841** magnetic nanoparticle solution or MNP into a cell suspension and gently mix with a pre-wet P1000 pipette tip.

2.6 2h

Incubate the suspension for 02:00:00 in a 37 °C incubator, 5 % CO_2. To ensure proper mixing during incubation, shake the tube on an orbital shaker at 250 rpm .

2.7 5m

After incubation, centrifuge the cell-MNP solution at 800 x g, 00:05:00 and remove the supernatant by pipetting.

2.8 Gently tap the cell pellet to resuspend the single cells and adjust the cell concentration to $1.33x10^5$ cells/mL by adding 2730 µL of expansion media.

2.9 Pipette the mixture up and down with a P1000 pipette tip to ensure a single cell suspension.

2.10 Place the ultra-low attachment 96 well plate on top of a 96-well spheroid magnetic drive prior to bioassembly and bioprinting (Figure 4A).

2.11

Transfer 150 µL of cell suspension to each well of the plate. To prevent aggregation of the magnetized cells, gently hand shake the tube during pipetting.

2.12 3h

Seal the border of the plate with 100 µL of sterilized water to minimize the evaporation of media and incubate the plate in 37 °C , 5 % CO_2 with humidity for 03:00:00 and observe cell morphology (Figure 4B).

2.13 1w 3d

Remove the magnetic drive from the bottom of the plate and incubate the plate further in 37 °C , 5 % CO_2 with humidity for 192:00:00 with medium replacement every 48:00:00 (Figure 4C).

2.14 1d

To induce cellular senescence, treat the organoids with 10 Milimolar (mM) - 100 Milimolar (mM) of Etoposide **Sigma-aldrich Catalog #E1383** in EEM at day 8 for 24:00:00 .

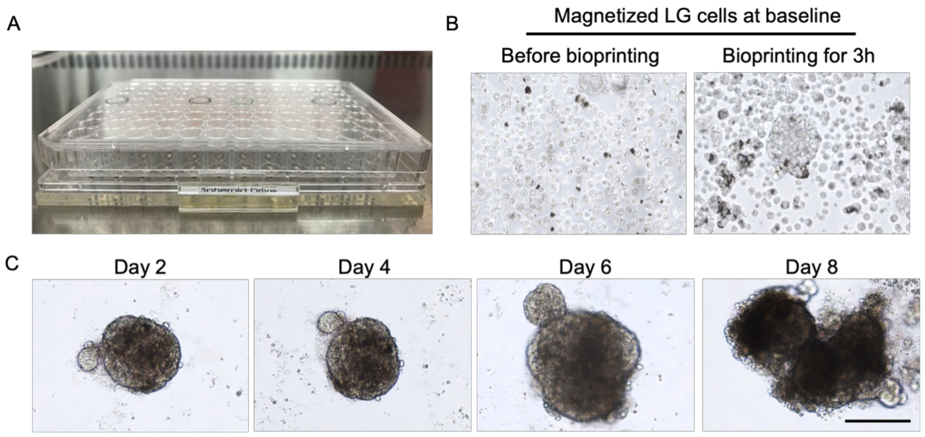

Figure 4. Magnetic 3D bioprinting and bioassembly. The organoids are biofabricated in each well at an ultra-low attachment 96 well plate with a magnetic spheroid driver underneath (A).Morphology of magnetized LG cells before and after bioassembly at baseline (B). After bioassembly, the SG organoid is cultured for 8 days (C). Scale bar: 200 µm.

3 Secretory LG/SG organoid validation

3.1 Immunofluorescent staining of acinar and ductal epithelial compartments 9h 30m

3.1 Use a magnetic holder to hold the organoid at the bottom of each well in the 96 well plate and remove all media from the organoids. Avoid sucking up the organoids and shear them through the P200 pipette tip (always use normal uncut tip for this step).

3.2 Gently wash the organoids with **200 µL** of 1X PBS and discard all solution.

3.3 30m

Fix the organoids by adding **100 µL** of **[M]4 Mass / % volume** Paraformaldehyde **Sigma Aldrich Catalog #P6148** (PFA) and incubate at room temperature for **00:30:00** with a **400 rpm** orbital swirling.

3.4 Use a magnetic holder to hold the organoid in each well of 96 well plate and remove all solutions (4% PFA and later 1X PBS) from the organoids.

3.5 Gently wash the organoids with **200 µL** of 1X PBS and discard all solution for three times.

If not used immediately, add **200 µL** of 1X PBS into each well and seal the plate with

parafilm. Store the plate in the fridge at 4 °C for up to 1 month.

3.6 For immunofluorescent labeling, remove residual 1X PBS with a P200 pipette tip as much as possible.

3.7 20m

Permeabilize the organoids with 200 µL of [M]0.1 % (v/v) Triton X for 00:20:00 with 400 rpm orbital swirling and then remove all solution with a P200 pipette tip.

3.8

Wash the organoids with 200 µL of 1X PBS and remove the solution with a P200 pipette tip for at least three times.

3.9 3h

Add 200 µL of blocking buffer into the center of organoids and incubate for 6 hours at room temperature or 01:00:00 - 02:00:00 at Room temperature followed by Overnight at 4 °C in a humidified chamber with a 400 rpm orbital shaker.

3.10 After incubation, remove the blocking buffer from organoids with a P200 pipette.

3.11 3h

Add 100 µL solution of primary antibodies (against protein markers of acinar and ductal epithelial cells) into the organoids and incubate for at least 03:00:00 at Room temperature or Overnight 4 °C in a humidified chamber with 400 rpm orbital shaker.

3.12 20m

After incubation, remove the solution from the organoids with a P200 pipette tip and wash the excess of antibody solution with 200 µL of [M]0.1 % (v/v) Tween-20 in 1X PBS for 00:20:00 with 400 rpm orbital shaker, at least three times.

3.13 1h

Add 100 µL of a solution with secondary antibodies into the organoids (specific to the host species of the previously used primary antibodies) and incubate at room temperature for 01:00:00 with a protection against photobleaching.

3.14 20m

After incubation, remove all solutions with secondary antibodies with a P200 pipette tip and rinse with 200 µL of a washing buffer solution containing [M]0.5 % (v/v) Tween-20 in 1X PBS for 00:20:00 with 400 rpm orbital shaker, at least three times.

3.15 1h

Replace the solution with 100 µL of nuclear stain solution ([M]10 % (v/v) Hoechst 33342 in 1X PBS) and incubate at Room temperature with 400 rpm orbital shaker for 01:00:00 .

3.16 Remove the nuclear stain solution and observe the labeled organoids under a fluorescence microscope before mounting them on a regular glass slide with a Fluoro-Gel III **Electron Microscopy Sciences Catalog #17985-50** resin mounting media.

Antibodies used

Antibodies	Source	Catalog number	Dilution
Rabbit monoclonal anti-AQP-5 IgG	Abcam	AB92320	1:100
Rabbit monoclonal anti-KRT14 IgG	Abcam	AB181595	1:100
Rabbit monoclonal anti-KRT19 igG	Novus Biologicals	NBP142238	1:100
Alexa Flour488 goat anti-rabbit IgG	Abcam	AB150077	1:200
Alexa Flour488 goat anti-mouse IgG	Abccam	150113	1:200

Antibodies

Solutions/media used

Collection media	Final concentrations	Volume (mL)
1XPBS	Not applicable	90
1XPBSPenicillin/Streptomycin (100%)	10% v/v	10
Total		100

Washing buffer	Final concentration	Volume (mL)
1XPBS	Not applicable	86.67
1XPBSPenicillin/Streptomycin (100%)	10%v/v	10
Bovine resum albumin (30%)	1%v/v	3.33
Total		100

Enzymatic dissociation buffer	Final concentration	Volume (mL)
1XPBS	Not applicable	1.763
1XPBSPenicillin/Streptomycin (100%)	1%v/v	0.020
Bovine resum albumin (30%)	1%v/v	0.067
Calcium chloride solution (50 mM)	1.25 mM	0.050
Collagenase II (40 mg/mL)	1 mg/mL	0.050
Hyaluronidase	1 mg/mL	0.050
Total		2

Basal media	Final concentration	Volume (mL)
DMEM/F12	Not applicable	98
L-Glutamine (100 mM)	1 mM	1
Penicillin/Streptomycin (100%)	1% v/v	1
Total		100

Expansion media (EM)	Final concentration	Volume (mL)
Basal media	Not applicable	94.85
Fetal bovine serum (100%)	5%v/v	5
EGF (20 µg/mL)	20 ng/mL	0.1
Total		100

Epithelial enrichment media (EEM)	Final concentration	Volume (mL)
Define Keratinocyte SFM	Not applicable	99.80
EGF (20 µg/mL)	20 ng/mL	0.1
FGF-10 (100 µg/mL)	50 ng/mL	0.05
FGF-7 (100 µg/mL)	50 ng/mL	0.05
Total		100

Blocking buffer	Final concentration	Volume (mL)
1XPBS	Not applicable	0.633
Fetal bovine serum (100%)	5%v/v	0.1
Horse serum (100%)	10%v/v	0.167
1% v/v Tween 20	0.1%v/v	0.1
Total		1

Nuclear stain buffer	Final concentration	Volume (mL)
1XPBS	Not applicable	900
Hoechst 33342 (100%)	10%v/v	100
Total		1
