# Supplementary figures and images for: Magnetic bioassembly platforms for establishing craniofacial exocrine gland organoids as aging in vitro models
Source: PLoS One. 2022 Aug 5;17(8):e0272644. doi: 10.1371/journal.pone.0272644 (PMC9355193; doi:10.1371/journal.pone.0272644)

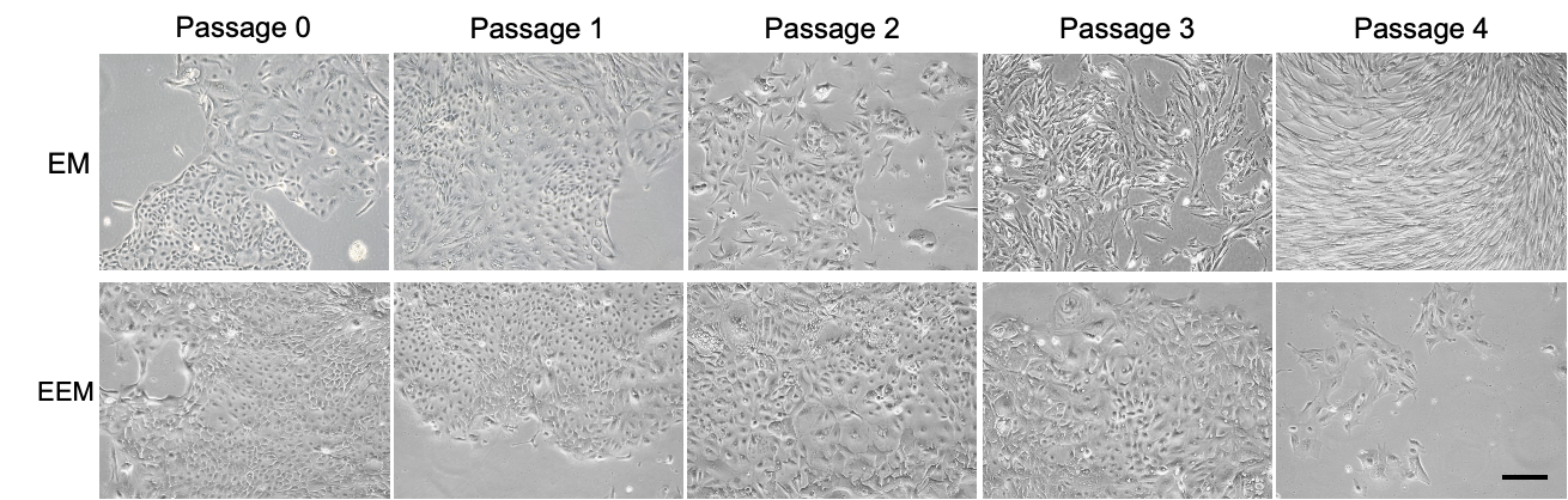

Supplement: S1 Fig — The morphology of LG cells in expansion media (EM) and epithelial enrichment media (EEM) up to passage 4. (TIF) [file pone.0272644.s003.tif]
