# Supplementary material for: Magnetic bioassembly platforms for establishing craniofacial exocrine gland organoids as aging in vitro models
Source: PLoS One. 2022 Aug 5;17(8):e0272644. doi: 10.1371/journal.pone.0272644 (PMC9355193; doi:10.1371/journal.pone.0272644)
Supplement: Attachment — Submitted filename: Response to reviewers R2.docx [file pone.0272644.s005.docx]

**Response to Reviewers – Manuscript** **PONE-D-22-06068R1**

July 21, 2022

**Dear PlosOne Editor and Reviewers**,

Thank you for giving us the opportunity to submit a revised version of the manuscript **PONE-D-22-06068R1** submitted to **PlosOne (Lab Protocol section)** and entitled **"Magnetic bioassembly platforms for establishing craniofacial exocrine gland organoids as aging in vitro models"**. Please take note that we have not been granted permission to change the title in the Protocols.io platform.

We appreciate the time and effort that you and the reviewers dedicated to providing feedback on our manuscript after our 1st revision and are grateful for the insightful comments.

In this letter, we provide a point-to-point reply in blue text to tackle the requested minor revisions:

**1) EDITOR COMMENTS & JOURNAL REQUIREMENTS:**

**1.1)** Please review your reference list to ensure that it is complete and correct. If you have cited papers that have been retracted, please include the rationale for doing so in the manuscript text, or remove these references and replace them with relevant current references. Any changes to the reference list should be mentioned in the rebuttal letter that accompanies your revised manuscript. If you need to cite a retracted article, indicate the article’s retracted status in the References list and also include a citation and full reference for the retraction notice.

**Authors response:** Thank you for checking those requirements. We have reviewed and edited the format of the reference list and have ensured that the reference format meets the PLOS ONE’s requirements. Please take note that we have corrected reference 45 for the tile and format since there is now a more recent revised version at bioRxiv. To our knowledge none of our cited papers have been retracted.

**REVIEW COMMENTS TO THE AUTHOR**

**2) REVIEWER #1:**

**2.1)** I thank the authors of the article for fulfilling the last minor issues. I accept this article for publication.

**Authors response:** Thank you for your input and for the time taken to review this manuscript.

**3) REVIEWER #2:**

**3.1)** Revisions to the figures are still required. Carbocol is still shown in figure 5 as 100 micromolar, even though the authors stated it is an error. Supplementary figure 2 showing spatial profiling in lung cancer still remains.

**Authors response:** Thank you for your feedback. We regret for the confusion caused. We may have accidentally uploaded the old figures (Fig 5 and S2 Figure were not correct). Please see the newly revised Figure 5 where we replaced "100 µM" carbachol with "10 µM". We also found one instance where carbachol concentration had a typo and we corrected it on the R2 revised version (see page 8 line 17). We also completely removed Supplementary Figure 2 from the submission platform as it was uploaded by mistake.

**3.2)** On the issue of differentiation, if all instances of "mature" differentiation or implication that the organoids are mature or fully differentiated are removed, then the AQP5 staining is fine and is informative as to the cell lineage. In the mouse, AQP5 is expressed in both embryonic "proacinar" cells as well as in adult acinar cells and so showing only AQP5 is insufficient to make a statement regarding maturity. There are insufficient studies in porcine tissue to know if its expression is similar.

**Authors response:** We agree with the reviewer's statement regarding the mouse AQP5 expression and the fact that there are insufficient studies for AQP5 in porcine salivary and lacrimal gland tissue. In this revised version, we did make sure that all instances of "mature" or "full" differentiation or the implications for such were completely removed (see page 9 line 15-16 and page 10 line 16-17).

**4) REVIEWER #3:**

**4.1)** The authors tried to meet the referees' queries sufficiently, and this revised article is worth publishing.

**Authors response:** Thank you for your feedback and for the time taken to review this manuscript.

We hope we have clarified the raised minor concerns with this newly revised version R2. Thank you again for your time and for considering this revised manuscript.

Sincerely,

João N. Ferreira, DDS MS PhD (Corresponding author on behalf of all authors)

E-mails: **Joao.F@chula.ac.th**
